# Physical Activity as a Lifestyle Modification in Patients With Multiple Comorbidities: Emphasizing More on Obese, Prediabetic, and Type 2 Diabetes Mellitus Patients

**DOI:** 10.7759/cureus.41356

**Published:** 2023-07-04

**Authors:** Arturo P Jaramillo, Sabina Ibrahimli, Javier Castells, Luisa Jaramillo, Denisse Moncada, Jhoanny C Revilla Huerta

**Affiliations:** 1 General Practice, California Institute of Behavioral Neurosciences & Psychology, Fairfield, USA; 2 Cardiology, Ivan Mikhailovich (IM) Sechenov First Moscow State Medical University, Moscow, RUS; 3 Medicine, Universidad Católica de Santiago de Guayaquil, Guayaquil, ECU; 4 Internal Medicine, Universidad Católica de Santiago de Guayaquil, Guayaquil, ECU; 5 Medicine, Universidad del Zulia, Maracaibo, VEN

**Keywords:** type 2 diabetes, prediabetic, intensive lifestyle change, exercise training, obese people

## Abstract

In this research, physical activity (PA) was shown to be inversely associated with the incidence of diabetes. This emphasizes the relevance of PA in diabetes prevention lifestyle intervention initiatives and encourages healthcare practitioners to advise high-risk patients on a healthy lifestyle combining PA for the reduction of weight in prediabetic, obese, and type 2 diabetes mellitus (T2DM) patients. The link between PA and diabetes was stronger in people with moderate or higher baseline PA, reflecting national recommendations that imply increasing activity levels may provide larger advantages for those who are comparatively less active.

An intensive lifestyle intervention of eating healthier foods and increasing PA resulted in an effective decrease in weight and waist circumference after one year, which has high potential in the long term to prevent T2DM and different comorbidities such as obesity. Studies such as PRomotion Of Physical activity through structured Education with differing Levels of ongoing Support for those with prediabetes (PROPELS) demonstrate that the combination of PA interventions with telemedicine follow-ups results in ambulatory activity changes in the first year, but these changes do not last longer than four years. Acute PA practicing regularly will reduce postprandial glucose excursions. However, it is unknown what type of PA routine will benefit the most from reducing postprandial glucose levels. There are no discernible variations in the effects of different disciplines of training on glucose levels, mainly when the data are compared across time. The combination of a healthy diet and lifestyle with programs based on diabetes prevention results in comparable and clinically significant mean weight reduction with cardiometabolic advantages. Based on the reviewed and cited studies, PA in patients at high risk of T2DM and obese and non-obese patients with T2DM results in favorable outcomes in the first few months. There is a large gap in investigations of the effects of PA in these patients and the benefits of other lifestyle modifications in long-term-based studies. However, in this study, we emphasize the importance of lifestyle modifications, putting in perspective principally the PA that the majority of patients with comorbidities do not practice, especially those with obesity, prediabetes, and T2DM. Thus, it would be necessary to conduct long-interval studies such as randomized clinical trials, where a better outcome can be given about intervals based on daily exercise times and the type of exercise in conjunction with diets that will have the greatest benefit, focusing more on the subjects that our research mentioned.

## Introduction and background

Lifestyle modifications are considered the best therapy for overweight patients. However, rigorous treatments such as the Diabetes Prevention Program [1] have been initially beneficial, but when the study population was retested in a randomized clinical trial, the programs often produced only a 2%-4% weight reduction.

From the prevention perspective, type 2 diabetes mellitus (T2DM) therapy minimizes the likelihood of serious consequences and permanent organ damage [2], demonstrating that exercise training is an essential part of overall diabetes treatment [3]. Physical activity (PA) is one of the four pillars of T2DM treatment. The Look AHEAD research participants attempted to lose more than 7% of their starting weight and increase moderately vigorous PA to more than three hours per week [4]. After 12 months, they showed improvements in their quality of life and a change in psychological outcome, lowering depression symptoms due to their physical appearance and motivation, and enhancing their physical endurance [5]. In T2DM patients, high levels of PA and participation in an organized exercise or sports program are associated with a higher quality of life. Aerobic-type exercise of adequate intensity is a significant factor in enhancing glucose absorption and tolerance as a form of T2DM prevention and treatment [6].

In a study on the impact of physical activity on the prevention of type 2 diabetes, after the diabetes prevention program (DPP) trial phase (3.2 years of average follow-up), the contributions of the two lifestyle objectives of weight reduction and physical activity (PA) were investigated in lifestyle participants. Those who did not fulfill the year one weight objective but did meet the expressed PA goal achieved a 46% reduced diabetes incidence after the DPP compared to those who met neither goal [7].

## Review

Methodology

A systematic review was conducted putting together free available full-length articles and utilizing the Preferred Reporting Items for Systematic Reviews and Meta-Analysis (PRISMA) checklist to document our methodology and conclusions. We have adhered to the PRISMA 2020 recommendations.

Study Duration

This review started on May 13, 2023.

Search Strategy

PubMed, Google Scholar, and Cochraine were used to collect database using the following: diabetes mellitus type 2 OR hyperglycemia OR insulin resistance OR (("Diabetes Mellitus, Type 2/diet therapy" (Majr) OR "Diabetes Mellitus, Type 2/prevention and control" (Majr) OR "Diabetes Mellitus, Type 2/therapy" (Majr))) OR ("Diabetes Mellitus, Type 2/diet therapy" (Majr:NoExp) OR "Diabetes Mellitus, Type 2/prevention and control"(Majr:NoExp) OR "Diabetes Mellitus, Type 2/therapy" (Majr:NoExp)) AND OBESITY OR OVERWEIGHT OR MORBID OBESITY OR (("Obesity/diet therapy" (Majr) OR "Obesity/prevention and control" (Majr) OR "Obesity/rehabilitation" (Majr) OR "Obesity/therapy" (Majr))) OR ("Obesity/diet therapy" (Majr:NoExp) OR "Obesity/prevention and control" (Majr:NoExp) OR "Obesity/rehabilitation" (Majr:NoExp) OR "Obesity/therapy"(Majr:NoExp)) AND AEROBIC EXERCISE OR CARDIOVASCULAR EXERCISE OR EXERCISE REGIMEN OR (("Exercise/physiology" (Majr) OR "Exercise/trends" (Majr))) OR ("Exercise/physiology" (Majr:NoExp) OR "Exercise/trends" (Majr:NoExp)). These terms were mixed using the Boolean operators and block approach, and "OR" and "AND" were used in combination with the appropriate keywords. The search results included free full-text articles and human trials in the English language.

Eligibility Criteria and Study Selection

To assess eligibility, two investigators carefully read the full title and content of each paper.

Inclusion and Exclusion Criteria

We selected the latest literature and articles published in the past two years, including papers written in the English language or if the full-text English language translation is available. We only included research papers involving human participants.

Articles were excluded if the full text of the papers could not be retrieved. Articles focused on lifestyle modification and physical activity in obese, prediabetic, and T2DM patients. Gray literature and proposal papers were also not included.

Data Management

Two independent writers evaluated papers based on titles and abstracts. Following that, significant abstracts were examined for complete full-text examination. The selected studies were evaluated, and if there was any dispute, the research was evaluated by a third author. Information from relevant publications was then collected. The first author’s name, research type, location, year of publication, sample size, and age were collected. Finally, duplicates were deleted.

Quality Assessment

For systematic reviews and meta-analyses, we used the assessment of multiple systematic review (AMSTAR) questionnaire and Cochrane risk of bias assessment tools for clinical trials.

Results

Search Results

**Figure 1:**
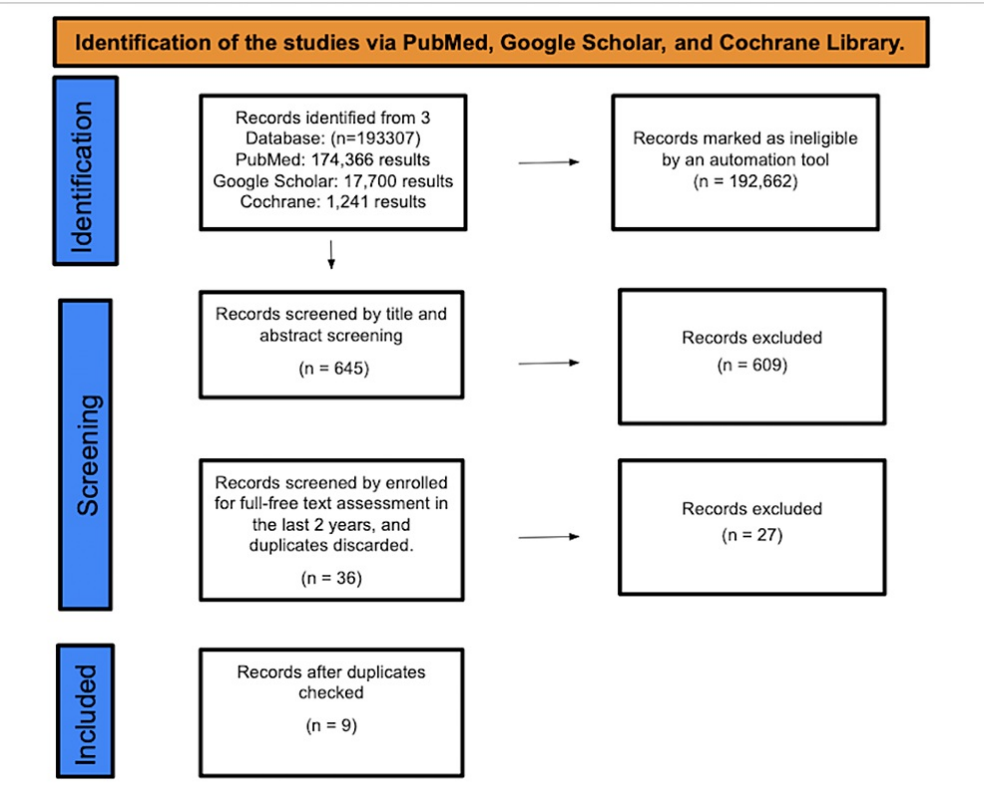
Identification of studies via databases and registers

A total of 193,307 studies were found after searching PubMed, Google Scholar, and Cochrane Library. A total of 192,662 were marked as ineligible by an automation tool. A total of 645 studies underwent title and abstract screening, and 609 papers were discarded. The remaining 36 papers were chosen for full-text evaluation in the previous two years, and after discarding duplicates, it resulted in the elimination of 27 studies. A total of nine studies were enlisted for the final collection of data (Figure 1).

Table 1 shows the summary and characteristics of all included studies.

**Table 1:** Table of data extraction RCT: randomized control trials, T2DM: type 2 diabetes mellitus, SRL: systematic review literature, AMSTAR: assessment of multiple systematic reviews, IG: intervention group, CG: control group, NAFLD: nonalcoholic fatty liver disease, PRT: progressive resistance training, WL: weight loss, BMI: body mass index, HbA1c: hemoglobin A1c, PA: physical activity, MetS: metabolic syndrome, WAP: walking away plus

Author	Year of publication	Study design	Quality tool	Primary research	Outcome evaluation
Fajriyah et al. [7]	2023	SRL	AMSTAR checklist	The 25 articles that met the inclusion criteria consisted of 18 randomized clinical trials and seven experiments.	There is an influence of land-based activity, aquatic physical activity, and physical endurance and depression on how patients with T2DM live.
Kriska et al. [8]	2021	RCT	Cochrane risk of bias assessment tool	There was a 6% decrease in the proportional hazard ratio. The p value of <0.001 showed how the incidence of diabetes decreased weekly during PA in the cohort after years.	PA was inversely related to the incidence of diabetes in the entire cohort.
Amin et al. [9]	2023	SRL	AMSTAR checklist	Eighty-seven patients were eligible for the study.	MetS classification reduced in >50% of participants in the IG and >80% in the CG (p < 0.05).
Freer et al. [10]	2022	RCT	Cochrane risk of bias assessment tool	During one year, weight loss with or without PRT showed a significant reduction in the fatty liver index, which indicates NAFLD.	In overweight adults with T2DM, PRT did not affect fatty liver index and weight loss benefit.
Yeung et al. [11]	2021	RCT	Cochrane risk of bias assessment tool	A total of 846 adults with impaired fasting glucose, isolated impaired glucose tolerance, or both were recruited.	A baseline weight loss of ≥5% and two hours per week of PA were the goals in the study.
Raben et al. [12]	2021	RCT	Cochrane risk of bias assessment tool	A total of 2,326 prediabetic patients with a BMI of ≥25 kg/m^2^ between 25 and 70 years old were selected for the study.	T2DM incidence after three years decreased. Diets, PA, and the combination of both had little effect.
Muilwijk et al. [13]	2021	RCT	Cochrane risk of bias assessment tool	A total of 3,684 women and men from Asia between 40 and 70 years old with no T2DM but elevated waist circumference and/or HbA1c were selected for this study.	At one year, the lifestyle adjustment administered by Asian health professionals to patients with elevated HbA1c and waist circumference was beneficial in lowering both waist circumference and weight.
Khunti et al. [14]	2021	RCT	Cochrane risk of bias assessment tool	Patients with risks of diabetes selected between 2013 and 2015 were randomized.	In one year, WAP raised ambulatory activity by 547 steps daily as opposed to control. WAP is also more likely to achieve 150 minute/week of moderate-to-vigorous PA.
Holzer et al. [15]	2021	RCT	Cochrane risk of bias assessment tool	In a random sequence, all individuals conducted three moderate exercise bouts: whole-body electromyostimulation, resistance exercise without electromyostimulation, and cycling endurance exercise.	Exercise reduced postprandial glucose in this research. This matches previous findings. Even at 20 minutes, all kinds of activity lowered glucose.
Das et al. [16]	2021	RCT	Cochrane risk of bias assessment tool	Group videoconferences with supplementary emails were held for healthy weight for living or diabetes prevention program participants.	The regimens used in this study had comparable and clinically significant effects on weight reduction and cardiometabolic improvements. These findings suggest a new obesity behavioral therapy.

Discussion

Aerobic Exercise

In men and women with T2DM, aerobic exercise and enhanced cardiorespiratory fitness reduce morbidity and mortality by 20% [16]. Aerobic exercise is a kind of physical activity that employs the aerobic energy-producing system to enhance capacity and efficiency and build cardiorespiratory endurance [7], and it uses vast muscle groups repeatedly [17]. It enhances insulin sensitivity, vascular compliance, lung function, and immunological function [18]. The effectiveness of PA in people with T2DM has shown a decrease in HbA1c, lipids, blood pressure, and insulin resistance [17].

In other studies, the timed up-and-go test and handgrip strength tests, which attest to the value of the prescribed protocol in this study’s intervention [19], showed that older women with T2DM had increased functional capacity after 10 weeks of aerobic training. Raben et al. [12] showed that after 36 months, patients on a high-protein diet with extenuating PA had a lower glycemic index (11.9%) compared to the other three groups (20%-21%). The body weight loss after two months was 11% and after 36 months was 5%, which was not much of a difference.

Amin et al. [9] found that a culturally relevant 12-week program tailored exclusively for the study population with T2DM improved the MetS score. A training program was used in which patients underwent a combination of aerobic and resistance exercises, and the findings indicated that the intervention group had a higher beneficial influence on the MetS severity score than the control group. The program used in patients with T2DM generated better results than aerobics or endurance exercise alone [20,21]. Furthermore, Pattyn et al. [22] concluded that aerobic, moderate, or high-intensity training will mitigate cardiovascular risk factors.

Wewege et al. [23] conducted a study to investigate how exercise helps reduce MetS markers in healthy individuals. There were 11 investigations in all, and patients’ lipid profiles, fast glycemic indexes, waist circumferences, and diastolic blood pressures improved significantly [23,24]. Most articles showed favorable effects on the MetS index, lipid levels, and glycemic risk factors; some studies reached therapeutic levels, and the psychological component of patients who were depressed due to being overweight or obese was influenced as well. A substantial part of this systematic review literature is to make known that there are still gaps in the research on the combination of physical disciplines to compare which is the best to prove the outcome of using them as therapies and preventive medicine in patients with T2DM, obesity, and prediabetes.

## Conclusions

Exercise affects type 2 diabetes patients’ physical stamina and quality of life. An aerobic training program may help with the treatment of type 2 diabetes, and it is a great option for this group because comorbidities such as obesity or lower limb dysfunction typically restrict patients from walking or jogging. Physical exercise on land and in water has substantial biological effects that extend beyond homeostatic systems and are therapeutically effective in the care of patients with musculoskeletal difficulties, cardiovascular illness, rheumatic disorders, and other ailments. In addition, one of the randomized clinical trials collected showed how the Ghanaian population PA regimen has the potential to improve MetS and quality of life in adults with T2DM.

New cases of T2DM will be significantly lower than expected three years after the simultaneous integration of diets, exercise, or both. This is based on maintaining a protein-based diet in conjunction with a continuous PA over three years, leading to favorable outcomes such as weight reduction and healthy food habits. All of these will lead to a 20% reduction in the incidence of T2DM. In older obese adults with T2DM, resistance training did not improve weight loss, a predictor of nonalcoholic fatty liver disease. Further large-scale studies to look for the benefits of aerobic training in patients with obesity, nonobese patients with T2DM, and those at risk of developing T2DM are needed to provide different insights about the possible synergistic effects of exercise and weight loss for the prevention of developing T2DM and as a first-line treatment in patients with T2DM.
